# Pandemic-related declines in hospitalization for non-COVID-19-related illness in the United States from January through July 2020

**DOI:** 10.1371/journal.pone.0262347

**Published:** 2022-01-06

**Authors:** Jennifer L. Nguyen, Michael Benigno, Deepa Malhotra, Farid Khan, Frederick J. Angulo, Jennifer Hammond, David L. Swerdlow, Maya Reimbaeva, Birol Emir, John M. McLaughlin

**Affiliations:** 1 Real World Evidence Center of Excellence, Pfizer Inc, New York, NY, United States of America; 2 Medical Development and Scientific/Clinical Affairs, Pfizer Vaccines, Pfizer Inc, Collegeville, PA, United States of America; 3 Clinical Development Internal Medicine and Hospital, Pfizer Global Product Development, Pfizer Inc, Collegeville, PA, United States of America; 4 Global Biometrics and Data Management, Pfizer Global Product Development, Pfizer Inc, Groton, CT, United States of America; 5 Global Biometrics and Data Management, Pfizer Global Product Development, Pfizer Inc, New York, NY, United States of America; Post Graduate Institute of Medical Education and Research, INDIA

## Abstract

**Background:**

The COVID-19 pandemic, caused by the novel severe acute respiratory syndrome coronavirus 2 (SARS-CoV-2), has substantially impacted healthcare utilization worldwide. The objective of this retrospective analysis of a large hospital discharge database was to compare all-cause and cause-specific hospitalizations during the first six months of the pandemic in the United States with the same months in the previous four years.

**Methods:**

Data were collected from all hospitals in the Premier Healthcare Database (PHD) and PHD Special Release reporting hospitalizations from January through July for each year from 2016 through 2020. Hospitalization trends were analyzed stratified by age group, major diagnostic categories (MDCs), and geographic region.

**Results:**

The analysis included 286 hospitals from all 9 US Census divisions. The number of all-cause hospitalizations per month was relatively stable from 2016 through 2019 and then fell by 21% (57,281 fewer hospitalizations) between March and April 2020, particularly in hospitalizations for non-respiratory illnesses. From April onward there was a rise in the number of monthly hospitalizations per month. Hospitalizations per month, nationally and in each Census division, decreased for 20 of 25 MDCs between March and April 2020. There was also a decrease in hospitalizations per month for all age groups between March and April 2020 with the greatest decreases in hospitalizations observed for patients 50–64 and ≥65 years of age.

**Conclusions:**

Rates of hospitalization declined substantially during the first months of the COVID-19 pandemic, suggesting delayed routine, elective, and emergency care in the United States. These lapses in care for illnesses not related to COVID-19 may lead to increases in morbidity and mortality for other conditions. Thus, in the current stage of the pandemic, clinicians and public-health officials should work, not only to prevent SARS-CoV-2 transmission, but also to ensure that care for non-COVID-19 conditions is not delayed.

## Introduction

The coronavirus disease 2019 (COVID-19) pandemic, caused by the novel severe acute respiratory syndrome coronavirus 2 (SARS-CoV-2), reached the United States in early 2020 [1]. Through the end of 2020, the Centers for Disease Control and Prevention (CDC) estimated that >83 million people had been infected with SARS-CoV-2 resulting in >4 million COVID-19 hospitalizations in the United States and >340,000 deaths [1,2].

The COVID-19 pandemic dramatically impacted healthcare utilization. COVID-19 was declared a national emergency in the United States in mid-March 2020 and state-based nonpharmaceutical mandates were implemented at around the same time [3,4]. Such state-mandated efforts to prevent SARS-CoV-2 transmission have included social-distancing measures, mask-wearing, stay-at-home orders, and recommendations to defer elective medical procedures [4,5]. Although limited regional data exist [6–8], no national studies have evaluated the impact of these SARS-CoV-2 mitigation efforts on overall trends in hospitalization. To understand the impact of the COVID-19 pandemic on overall US hospitalization trends, this study compared trends in all-cause and cause-specific hospitalizations during the first six months of the COVID-19 pandemic in the United States in 2020 with the same months in 2016–2019. Such information can inform regional and national public health officials and healthcare professionals of the ongoing concerns regarding non-COVID-19-related healthcare utilization during the pandemic and the need for appropriate public health messaging regarding the importance of seeking timely care.

## Methods

### Study population and outcomes

This retrospective analysis utilized the hospital discharge Premier Healthcare Database (PHD; Charlotte, NC, USA) as well as a Special Release of the PHD (PHD-SR) specifically developed for COVID-19 research [9,10]. The PHD is a hospital-based, service-level, all-payer database containing administrative information on inpatient discharges from nongovernmental, community, and teaching hospitals and health systems from urban and rural areas located in all 9 US Census divisions [11]. The PHD includes approximately 25% of all acute (i.e., emergency, urgent, trauma, and newborn) and elective hospital admissions in the US, and most hospitals included are typically large (>200 beds), allowing for a greater sampling percentage. Premier employs quality assurance procedures and undertakes routine audits to ensure the accuracy of information in the PHD. All data are deidentified and fully compliant with the Health Insurance Portability and Accountability Act (HIPAA). The PHD-SR contains data from a subset of the facilities included in the PHD that submit data on a daily, weekly, biweekly (every-two weeks), or monthly basis with an approximate data lag time of 1 to 3 weeks from date of discharge [9]. Data were extracted from PHD for 2016–2019, and PHD-SR for 2020. This study followed the Strengthening the Reporting of Observational Studies in Epidemiology (STROBE) reporting guidelines [12]. Given the exclusive use of deidentified data, this study was deemed not to require ethics board review based on the policy of the Office of Human Subjects Research Protections, National Institutes of Health, under the revised Common Rule.

**Fig 1:**
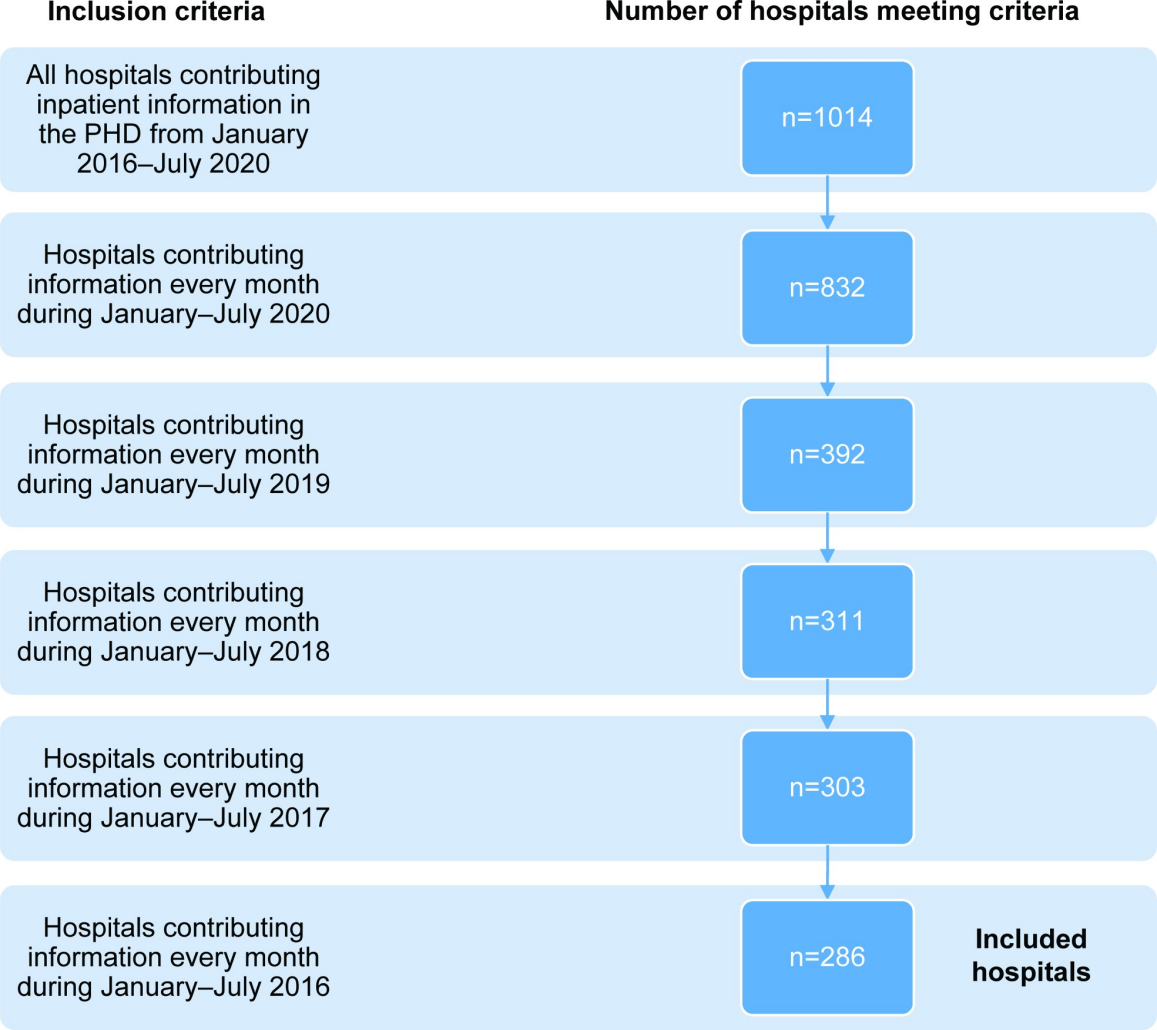
Hospital selection criteria. PHD = Premier Healthcare Database.

All hospitals in PHD that consistently reported hospitalization data for January through July of each year from 2016 to 2020 were included in the analysis (**Fig 1**). Hospitalizations were stratified by age, major diagnostic categories (MDCs), and COVID-19 diagnosis. The MDCs consist of 25 mutually exclusive principal diagnostic categories that generally correspond to the major organ system involved and associated medical specialty [13]. These standardized categories are developed by physician panels to provide a high-level classification hierarchy used by the US Medicare’s hospital reimbursement system [13].

COVID-19 was identified by a primary or secondary International Classification of Diseases Tenth Revision (ICD-10) diagnosis code of U07.1, which came into use on April 1, 2020 [14]. If diagnosis code U07.1 was reported as the principal diagnosis, the hospitalization was classified into one of three MDCs: Respiratory System, Newborn and Other Neonates, or Human Immunodeficiency Virus (HIV) Infection; hospitalizations with a secondary diagnosis of U07.1 could be classified into any MDC [15]. US Census division, hospital setting (urban or rural), hospital academic status (teaching or nonteaching), and number of beds were also available. The primary outcome of interest was the number of all-cause hospitalizations per month, which was stratified by Respiratory versus non-Respiratory System MDC, age group (0–17, 18–49, 50–64, ≥65 years), US Census division (East North Central, East South Central, Middle Atlantic, Mountain, New England, Pacific, South Atlantic, West North Central, West South Central) [16], and all 25 MDCs. We hypothesized that the number of all-cause hospitalizations between January and July of 2020 would be different from the average all-cause hospitalizations between 2016 and 2019.

### Statistical analysis

The average number of hospitalizations per month for the time period 2016–2019 were compared to the number of hospitalizations per month in 2020 using descriptive statistics (SAS version 9.4, SAS Institute, Cary, NC, USA).

## Results

### Overview of hospitals included in analysis

Of 1,014 hospitals captured in PHD from 2016 to July 2020, 286 (28%) had information for January through July for all five study years (2016 through 2020) and were included in the final analysis. The included hospitals were located in all 9 US Census divisions, however the number of hospitals per division was not necessarily proportional to the total number of hospitals in PHD per division (**Fig 1**; **Table 1**). Most of the included hospitals were in an urban region (69%; n = 196) and were nonteaching hospitals (69%; n = 198). Greater than half of included hospitals had >200 beds (52%). Overall, 78 hospitals (27%) had 0 to 99 beds and 60 (21%) had 100 to 199 beds (**Table 1**). The geographic distribution of included hospitals by Census division ranged from 6 hospitals (2%) in the New England division to 82 (29%) hospitals located in the South Atlantic division (**Table 1**). There were 63,411 COVID-19 hospitalizations between April and July 2020 among the study hospitals.

**Table 1:** Characteristics of study hospitals by Census division, January–July in 2016–2020.

	New England	Middle Atlantic	South Atlantic	East South Central	West South Central	East North Central	West North Central	Mountain	Pacific	All Hospitals
US states	Maine, Vermont, New Hampshire, Massachusetts, Connecticut, Rhode Island	New York, Pennsylvania, New Jersey	Delaware, Maryland, West Virginia, Virginia, North Carolina, South Carolina, Georgia, Florida, District of Columbia	Kentucky, Tennessee, Mississippi, Alabama	Oklahoma, Arkansas, Louisiana, Texas	Wisconsin, Michigan, Illinois, Indiana, Ohio	North Dakota, South Dakota, Minnesota, Iowa, Nebraska, Kansas, Missouri	Montana, Idaho, Wyoming, Nevada, Utah, Colorado, Arizona, New Mexico	Washington, Oregon, California, Alaska, Hawaii	
Hospitals contributing information into PHD, n (%)^a^	21 (2)	116 (11)	229 (23)	90 (9)	112 (11)	187 (18)	75 (7)	54 (5)	130 (13)	1014
Included hospitals (meeting study criteria), n (%)	6 (2)	37 (13)	82 (29)	17 (6)	25 (9)	46 (16)	16 (6)	29 (10)	28 (10)	286
Regional status, n (%)										
Urban	5 (83)	30 (81)	50 (61)	7 (41)	22 (88)	34 (74)	8 (50)	20 (69)	20 (71)	196 (69)
Rural	1 (17)	7 (19)	32 (39)	10 (59)	3 (12)	12 (26)	8 (50)	9 (31)	8 (29)	90 (31)
Academic status, n (%)										
Teaching	3 (50)	25 (68)	18 (22)	3 (18)	4 (16)	19 (41)	6 (38)	4 (14)	6 (21)	88 (31)
Nonteaching	3 (50)	12 (32)	64 (78)	14 (82)	21 (84)	27 (59)	10 (63)	25 (86)	22 (79)	198 (69)
Beds, n (%)										
0–99	1 (17)	4 (11)	24 (29)	2 (12)	10 (40)	9 (20)	6 (38)	12 (41)	10 (36)	78 (27)
100–199	2 (33)	8 (22)	20 (24)	3 (18)	1 (4)	10 (22)	4 (25)	5 (17)	7 (25)	60 (21)
200–299	0 (0)	9 (24)	14 (17)	3 (18)	3 (12)	11 (24)	0 (0)	3 (10)	3 (11)	46 (16)
300–399	0 (0)	5 (14)	9 (11)	5 (29)	5 (20)	4 (9)	1 (6)	4 (14)	4 (14)	37 (13)
400–499	1 (17)	2 (5)	2 (2)	2 (12)	0 (0)	6 (13)	3 (19)	1 (3)	2 (7)	19 (7)
≥500	2 (33)	9 (24)	13 (16)	2 (12)	6 (24)	6 (13)	2 (13)	4 (14)	2 (7)	46 (16)

PHD = Premier Healthcare Database.

^a^Percentages may not sum up to 100% due to rounding.

### All-cause hospitalizations

**Fig 2:**
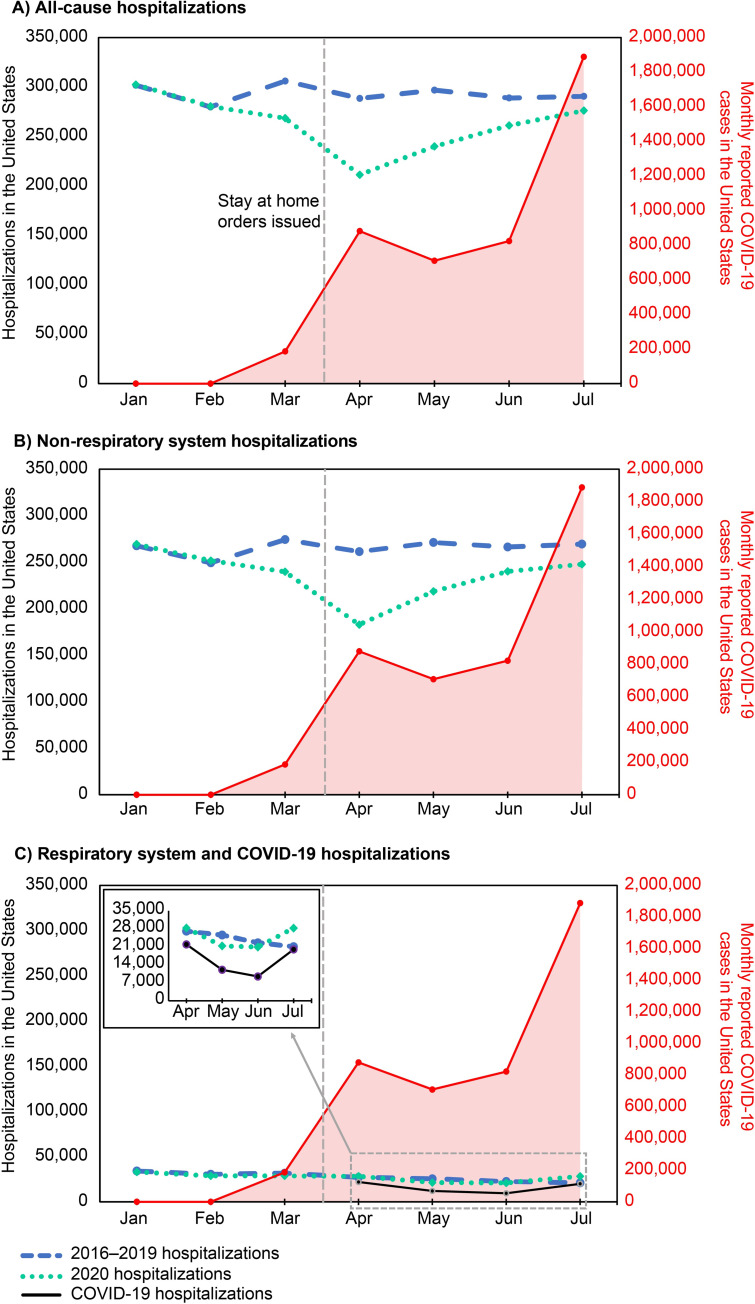
Average monthly hospitalizations, January-July, 2016–2019 compared to monthly hospitalizations and monthly reported COVID-19 cases* in 2020, United States. (**A**) All-cause hospitalizations, (**B**) Non–respiratory system hospitalizations, (**C**) Respiratory system and COVID-19 hospitalizations. Dashed blue line, 2016–2019 hospitalizations; dotted green line, 2020 hospitalizations; solid red line (right-side y-axis), COVID-19 cases; solid black line, COVID-19 hospitalizations; vertical black dotted line, when state-mandated social distancing including stay-at-home orders began (mid-March 2020). COVID-19 = coronavirus disease 2019. *COVID-19 case counts were extracted from the COVID-19 Dashboard by the Center for Systems Science and Engineering at Johns Hopkins University [17].

The average number of all-cause hospitalizations per month was generally stable during 2016–2019 (**Fig 2A; S1 Table**). In contrast, there was a 21% decrease in the number of all-cause hospitalizations per month between March and April 2020 (57,281 fewer all-cause hospitalizations in April), coinciding with the declaration of a national emergency in mid-March [3] (**Fig 2A**). From April onward there was a rise in the number of all-cause hospitalizations per month (**Fig 2A**). The decline in hospitalizations per month was predominantly among non-respiratory system hospitalizations (**Fig 2B**). There was no decline in hospitalizations per month among respiratory system hospitalizations observed during this same period (March to April 2020; **Fig 2C**). Following this, there was a 26% decline in respiratory system hospitalizations per month from April to June 2020 (7386 fewer hospitalizations in June), followed by a 37% increase from June to July 2020 (7821 more hospitalizations in July; **Fig 2C**). This trend mirrored monthly COVID-19 hospitalization trends during the same time (**Fig 2C**). Although increases in average monthly all-cause and non-respiratory hospitalizations were observed from April to July 2020, these increases were not proportional to the large increases in the monthly number of COVID cases observed from May to July 2020 (**Fig 2A and 2B; S1 Table**). Similarly, changes in the average monthly respiratory system and COVID-19 hospitalizations in 2020 did not parallel the overall increases in monthly reported COVID-19 cases (**Fig 2C; S1 Table**).

### Hospitalizations by major diagnostic category

**Fig 3:**
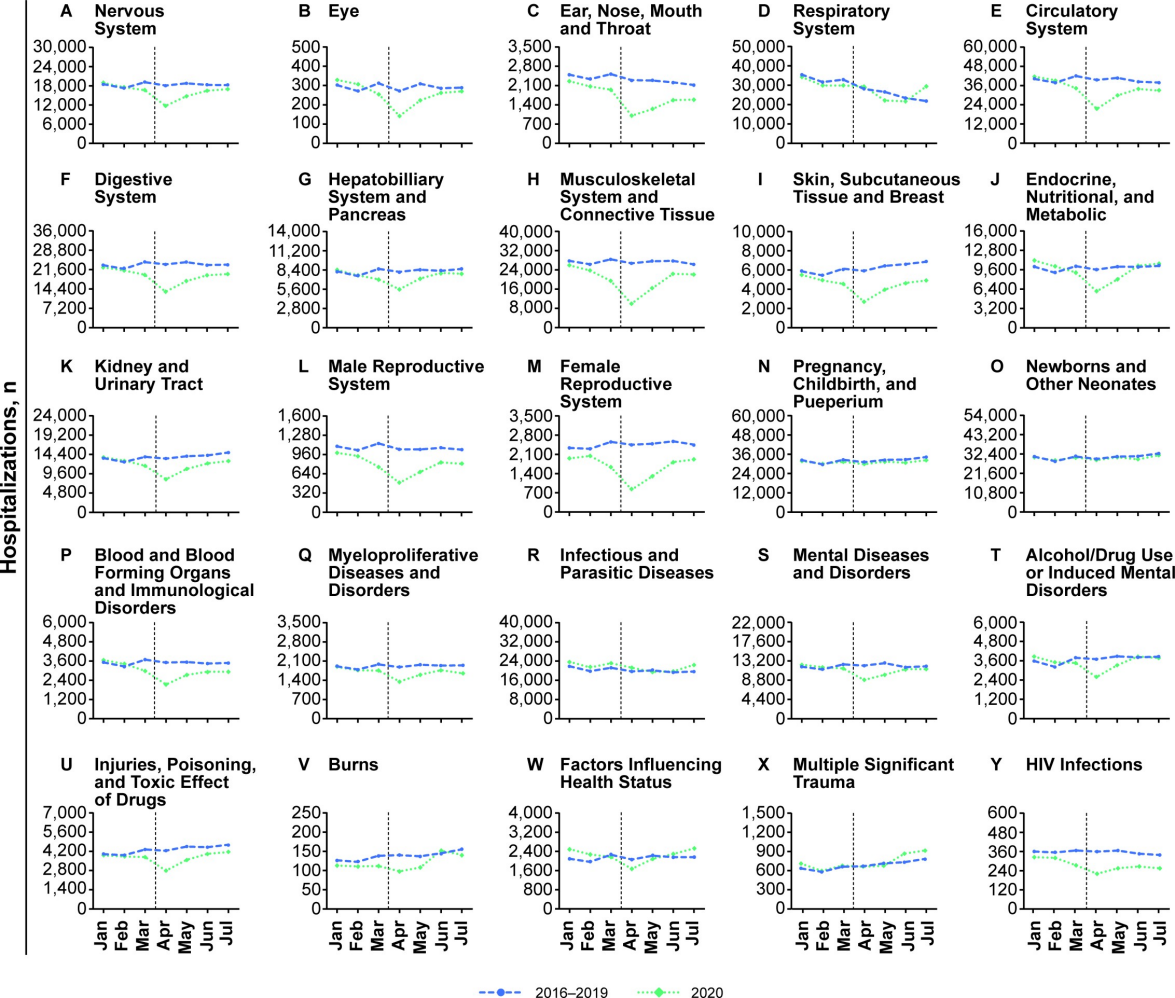
Average monthly hospitalizations, January-July, in 2016–2019 compared with monthly hospitalizations in 2020, by major diagnostic category, United States. Dashed blue line, 2016–2019 hospitalizations; dotted green line, 2020 hospitalizations; vertical black dotted line, when stay-at-home orders began (mid-March 2020). COVID-19 = coronavirus disease 2019.

Overall, average monthly hospitalizations by MDC from January to July were significantly different for 2020 compared with 2016–2019, with the exception of Multiple Significant Trauma (*P* = 0.1517; **S2 Table**). There was a decrease in the number of hospitalizations per month between March and April 2020, relative to the same period in 2016–2019, for 20 of the 25 MDCs (**Fig 3; S2 Table**). Monthly hospitalizations for these MDCs increased after April 2020, occasionally reaching the same levels as those observed in 2016–2019. The two MDCs which had no decline in monthly hospitalizations were *Pregnancy*, *Childbirth*, *and Puerperium* and *Newborns and Other Neonates*. These MDCs had similar monthly hospitalizations in 2020 as in 2016–2019 (approximately 30,000 per month for both; **Fig 3**). Five MDCs had an increase in monthly hospitalizations in 2020 compared to 2016–2019: *Respiratory System*; *Infectious and Parasitic Diseases*; *Multiple Significant Trauma*; *Endocrine*, *Nutritional*, *and Metabolic*; and *Factors Influencing Health Status* (**Fig 3**).

The trends in monthly hospitalizations by MDC for each Census division were generally similar to those observed nationwide (**S1–S9 Figs**). In the Middle Atlantic, monthly hospitalizations for the two MDCs, *Respiratory System* and *Infectious and Parasitic Diseases*, increased between March and April in 2020, after which they subsequently fell below the levels seen in 2016–2019 (**S3 Fig**). In contrast, in the South Atlantic, East South Central, West South Central, and Mountain regions, monthly hospitalizations for the *Respiratory System* MDC increased in July 2020 and were higher than numbers reported during the same timeframe for 2016–2019 (**S3–S5 and S8 Figs**). Similarly, increases in monthly hospitalizations for *Infectious and Parasitic Diseases* were observed in the South Atlantic, West South Central, and Mountain regions in June and July 2020 (**S3, S5 and S8 Figs**).

### All-cause hospitalizations by age group

**Fig 4:**
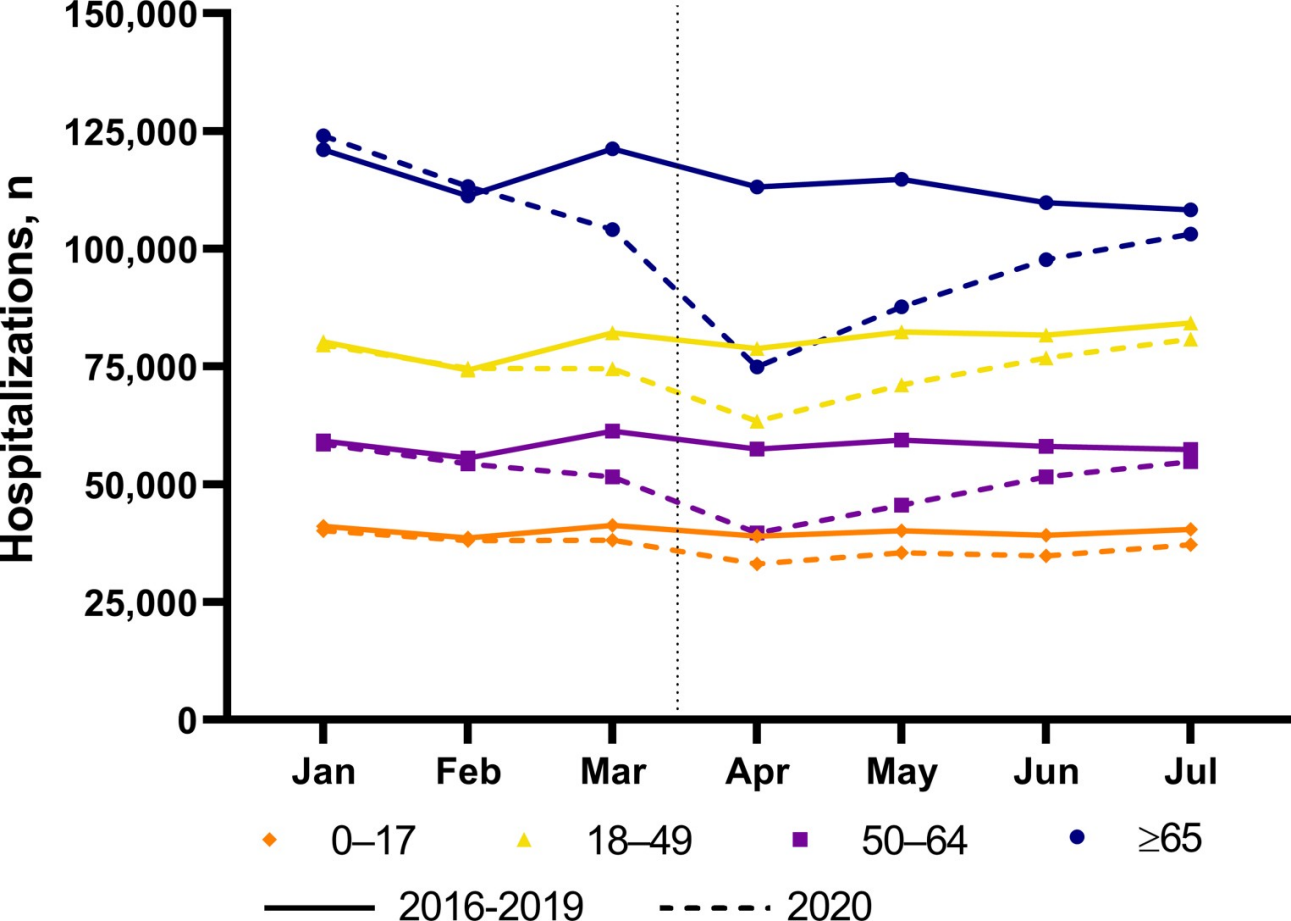
Average monthly all-cause hospitalizations, January-July, in 2016–2019 compared with monthly all-cause hospitalizations in 2020, by age group, United States. Vertical black dotted line, when stay-at-home orders began (mid-March 2020). COVID-19 = coronavirus disease 2019.

Across all five study years, the number of hospitalizations per month were highest for those aged ≥65 years, followed by the 18- to 49-year age group and the 50- to 64-year age group (**Fig 4; S3 Table**). Monthly hospitalizations were lowest among those 0–17 years of age. For all age groups, monthly hospitalizations were stable from January through July 2016–2019 (**Fig 4**). In contrast, there was a decrease in the monthly hospitalizations for all age groups between March and April, 2020 (0–17 years, 13% decline; 18–49 years, 15% decline; 50–64 years, 23% decline; and ≥65 years, 28% decline), after which there was a steady increase in the number of monthly hospitalizations until the end of the analysis period (July 2020; **Fig 4**). By July 2020, the number of hospitalizations per month for all age groups had almost returned to levels observed during the 2016–2019 period.

Trends in monthly all-cause hospitalizations for all age groups in all Census divisions mirrored those observed on the national level for the 2016–2019 and 2020 periods (**S10 Fig**). Notably, in the Middle Atlantic division, monthly hospitalization rates for all age groups remained lower in July 2020 compared with July in 2016–2019.

## Discussion

This study describes an important decline in hospitalizations during the first six months of the COVID-19 pandemic in the United States compared with the previous four years. Monthly all-cause hospitalizations fell by 21% between March and April 2020 following the state-based implementation of nonpharmaceutical interventions, including stay-at-home orders and social distancing mandates, aimed at mitigating SARS-CoV-2 transmission. The decline in monthly hospitalizations was largely attributed to a decline in monthly non-respiratory hospitalizations. Monthly respiratory hospitalizations increased between March and April in 2020 compared with prior years. This overall decline in monthly hospitalizations was consistent across all Census divisions in the United States and for the majority of non-respiratory MDCs, with the exception of pregnancy-related discharges, suggesting a nationwide trend. Pregnancy-related hospitalizations reflect a category of care that, largely, cannot be avoided suggesting the only admission categories not impacted by the pandemic were those that were absolutely necessary.

The decline in hospitalizations in the United States is likely due to patients avoiding seeking medical care for urgent conditions, routine care, and elective procedures during the early stages of the COVID-19 pandemic. In this study, the decline observed in hospitalizations occurred for the majority of MDCs. Several studies have reported reductions in ER visits for serious surgical and medical diagnoses [6,7,18–21] as well as reductions in overall healthcare utilization and expenditure [22] during the course of the COVID-19 pandemic. In a survey by the CDC, approximately 41% of US adults reported that they had delayed or avoided medical care during the COVID-19 pandemic, including 12% of adults who reported not seeking emergency care [18]. In this study, the reduction in hospitalizations between March and April 2020 after stay-at-home orders began compared with the same timeframe in 2016–2019 were more dramatic for certain MDCs (eg, *Circulatory System*; *Digestive System*; *Kidney and Urinary Tract*; *Endocrine*, *Nutritional*, *and Metabolic System*; *Musculoskeletal System*; *Hepatobiliary System and Pancreas*) than others. These MDCs include acute diagnoses, such as myocardial infarction, stroke, appendicitis, pancreatitis, and cirrhosis among others, and are typically associated with the need for emergency care. Because sudden largescale shifts in the incidence of these acute conditions in the United States and globally are unlikely, such observations suggest that patients were avoiding hospitals, likely because of fear of contracting COVID-19. Although hospitalization numbers have somewhat rebounded since the easing of lockdown measures, they were still below historical levels in July, suggesting that people were continuing to avoid seeking care. Not seeking timely care for conditions requiring prompt attention could lead to worsened patient outcomes. Moreover, missing routine care appointments could lead to missed opportunities for the management of chronic conditions.

Hospitalization trends across US Census divisions largely mirrored those seen on a national scale, with some exceptions. For example, in the Middle Atlantic division, which includes New York and New Jersey, monthly hospitalizations for the two MDCs, *Respiratory System* and *Infectious and Parasitic Diseases*, increased between March and April in 2020, after which they subsequently fell below the levels seen in 2016–2019. It is likely that a combination of residents leaving the New York-New Jersey area amid the pandemic and stringent and extended lockdown measures led to decreased hospitalizations for these two MDCs during the latter stages of the analysis period [23,24]. In contrast, hospitalizations for these two MDCs increased in July 2020 compared with July in 2016–2019 in several Census divisions, including the South Atlantic, East South Central, West South Central, and Mountain divisions. The geographic regions most affected by COVID-19 outbreaks have shifted over time. During the early stages of the pandemic, New York City and other coastal regions were hotspots for the COVID-19 outbreak followed by a spread inwards to other parts of the United States [25,26]. Thus, it is unsurprising to see differences in the timing of hospitalization trends between different Census Divisions over the course of the study. Additionally, as many states within the South Atlantic, East South Central, West South Central, and Mountain divisions began lifting stay-at-home orders during the later parts of the study period, this may have also led to greater spread of SARS-CoV-2.

Reductions in the number of hospitalizations by age group during stay-at-home orders were most dramatic for adults aged 50 to 64 years and ≥65 years. In contrast, the reductions in hospitalization numbers were less pronounced for those <49 years old. These trends were reflected across most Census divisions. These differences could be attributable to different mitigation behaviors by age group. Multiple surveys by the CDC between April and June 2020 observed that older age was positively associated with greater COVID-19 mitigation behaviors, including mask-wearing, maintaining social distance, and avoiding public or crowded places [27]. These surveys consistently reported that adults aged ≥60 years took greater precautions compared with those aged 18 to 29 years [27]. These observations, taken together with higher risk for COVID-19 mortality in the elderly [28], suggests that individuals in this older age group may have postponed routine or urgent care during the stay-at-home orders. This is supported by data showing an increase in out-of-hospital cardiac arrests in 2020 compared with 2019, especially among the elderly and among those with comorbidities like diabetes and hypertension [29], confirming that avoidance of critical care needs by the elderly during the pandemic may result in negative outcomes.

Current evidence also suggests a decline in quality of life and lowered mental health during the pandemic. A nationwide survey of 6000 adults reported that overall alcohol consumption increased up to 14% between May 28 and June 16, 2020, compared with data collected during the same timeframe in 2019 [30]. Additionally, studies have reported deteriorating mental health conditions among the general population as well as healthcare professionals involved in delivering COVID-19–related care during the pandemic [31–33]. One study suggested that the prevalence of depression symptoms had increased 3-fold in the United States during this time compared with pre-pandemic times [31]. In agreement with these findings, this study showed that hospitalizations for *Alcohol/Drug Use or Induced Mental Disorders* began to trend higher than historical levels by July 2020 in some Census divisions.

Since the lifting of lockdown measures, certain ongoing surveys have suggested that hospital utilization may be increasing but continues to remain below pre-pandemic levels [34]. Overall, decreases in hospital utilization and visits for acute illnesses during the pandemic could have negative implications for the health and wellness of the general population. Additionally, high-risk and vulnerable populations may be particularly susceptible to poor health outcomes because of delayed care [28,35–37]. Accordingly, the negative effects of underutilization of healthcare may have long-term consequences that extend beyond the end of the pandemic.

This study has certain limitations including those related to administrative data that must be considered when interpreting the results. Although the PHD includes large hospitals and captures approximately 25% of all US hospital admissions, data were available for only 28% of the 1014 hospitals captured in PHD after restricting the study to hospitals consistently reporting data for January through July from 2016 to 2020. Thus, the trends observed in this study may not be representative of the entire US. Moreover, the identification of COVID-19 cases relied on discharge coding accuracy. Nevertheless, a study using the PHD database concluded that the use of the ICD-10 diagnosis code U07.1 was widely adopted across hospitals early on during the pandemic and has been reasonably accurate for tracking COVID-19 hospitalizations [38]. Another limitation is that the reasons for the observed decline in hospitalizations during the pandemic could not be comprehensively explored. Temporally, the declines in hospitalization occurred during times of stringent state-based mandates for social distancing including stay-at-home orders. However, it may be likely that the dominant reason for the decline in hospitalizations is due to patient’s deferral of healthcare services during the pandemic [39]. Notably, increased social distancing may have also resulted in declines in the incidence of some non-COVID-19 diseases (such as influenza or other respiratory pathogens) that also may have contributed to the decline in hospitalizations during the pandemic. Further, this study only examined the impact of the pandemic on hospitalizations, and did not examine telehealth or outpatient visits, which may have replaced or offset some of the healthcare utilization for less serious conditions and categories. Additional studies are needed to determine if reductions in hospitalizations were more prevalent in certain at-risk groups and if these could lead to long-term effects even after the end of the pandemic.

## Conclusions

During the first six months of the COVID-19 pandemic in the United States, monthly hospitalizations declined dramatically compared with the previous four years, suggesting that many people in the United States delayed both routine and emergency care. Lapses in care for serious or life-threatening conditions could lead to eventual increases in morbidity and mortality for illnesses not related to COVID-19. Thus, the health effects of the COVID-19 pandemic most likely extend beyond those related directly to SARS-CoV-2 infection, and more individual-level data describing the secondary effects of the pandemic are needed. In the current stage of the pandemic, clinicians and public-health officials should work not only to prevent SARS-CoV-2 transmission but also to ensure that care for non-COVID-19 conditions is not delayed.

## Supporting information

S1 Fig Average monthly hospitalizations, January-July, in 2016–2019 compared with monthly hospitalizations in 2020, by major diagnostic category in the New England Census division. (TIF)

S2 Fig Average monthly hospitalizations, January-July, in 2016–2019 compared with monthly hospitalizations in 2020, by major diagnostic category in the Middle Atlantic Census division. (TIF)

S3 Fig Average monthly hospitalizations, January-July, in 2016–2019 compared with monthly hospitalizations in 2020, by major diagnostic category in the South Atlantic Census division. (TIF)

S4 Fig Average monthly hospitalizations, January-July, in 2016–2019 compared with monthly hospitalizations in 2020, by major diagnostic category in the East South Central Census division. (TIF)

S5 Fig Average monthly hospitalizations, January-July, in 2016–2019 compared with monthly hospitalizations in 2020, by major diagnostic category in the West South Central Census division. (TIF)

S6 Fig Average monthly hospitalizations, January-July, in 2016–2019 compared with monthly hospitalizations in 2020, by major diagnostic category in the East North Central Census division. (TIF)

S7 Fig Average monthly hospitalizations, January-July, in 2016–2019 compared with monthly hospitalizations in 2020, by major diagnostic category in the West North Central Census division. (TIF)

S8 Fig Average monthly hospitalizations, January-July, in 2016–2019 compared with monthly hospitalizations in 2020, by major diagnostic category in the Mountain Census division. (TIF)

S9 Fig Average monthly hospitalizations, January-July, in 2016–2019 compared with monthly hospitalizations in 2020, by major diagnostic category in the Pacific Census division. (TIF)

S10 Fig Average monthly all-cause hospitalizations, January-July, in 2016–2019 compared with monthly all-cause hospitalizations in 2020, by age group and Census division, United States. (TIF)

S1 Table Average monthly hospitalizations, January–July 2016–2019 compared with monthly hospitalizations and monthly reported COVID-19 cases* in 2020, United States. *COVID-19 case counts were extracted from the COVID-19 Dashboard by the Center for Systems Science and Engineering at Johns Hopkins University [17].
(DOCX)

S2 Table Average monthly hospitalizations, January–July, in 2016–2019 compared with monthly hospitalizations in 2020, by major diagnostic category, United States. (DOCX)

S3 Table Average monthly all-cause hospitalizations, January–July, in 2016–2019 compared with monthly all-cause hospitalizations in 2020, by age group, United States. (DOCX)
