# Supplementary figures and images for: Pandemic-related declines in hospitalization for non-COVID-19-related illness in the United States from January through July 2020
Source: PLoS One. 2022 Jan 6;17(1):e0262347. doi: 10.1371/journal.pone.0262347 (PMC8735608; doi:10.1371/journal.pone.0262347)

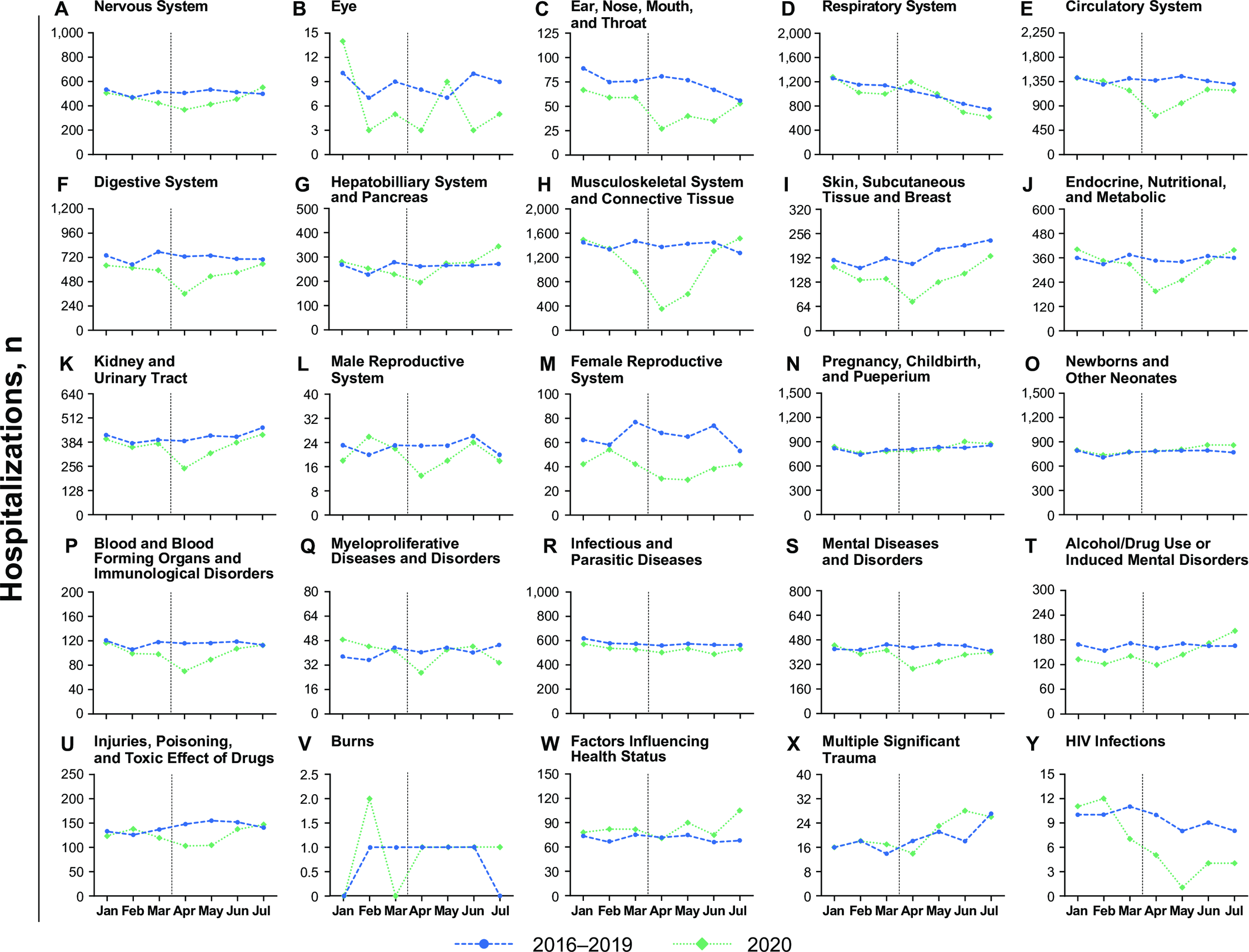

Supplement: S1 Fig — (TIF) [file pone.0262347.s001.tif]

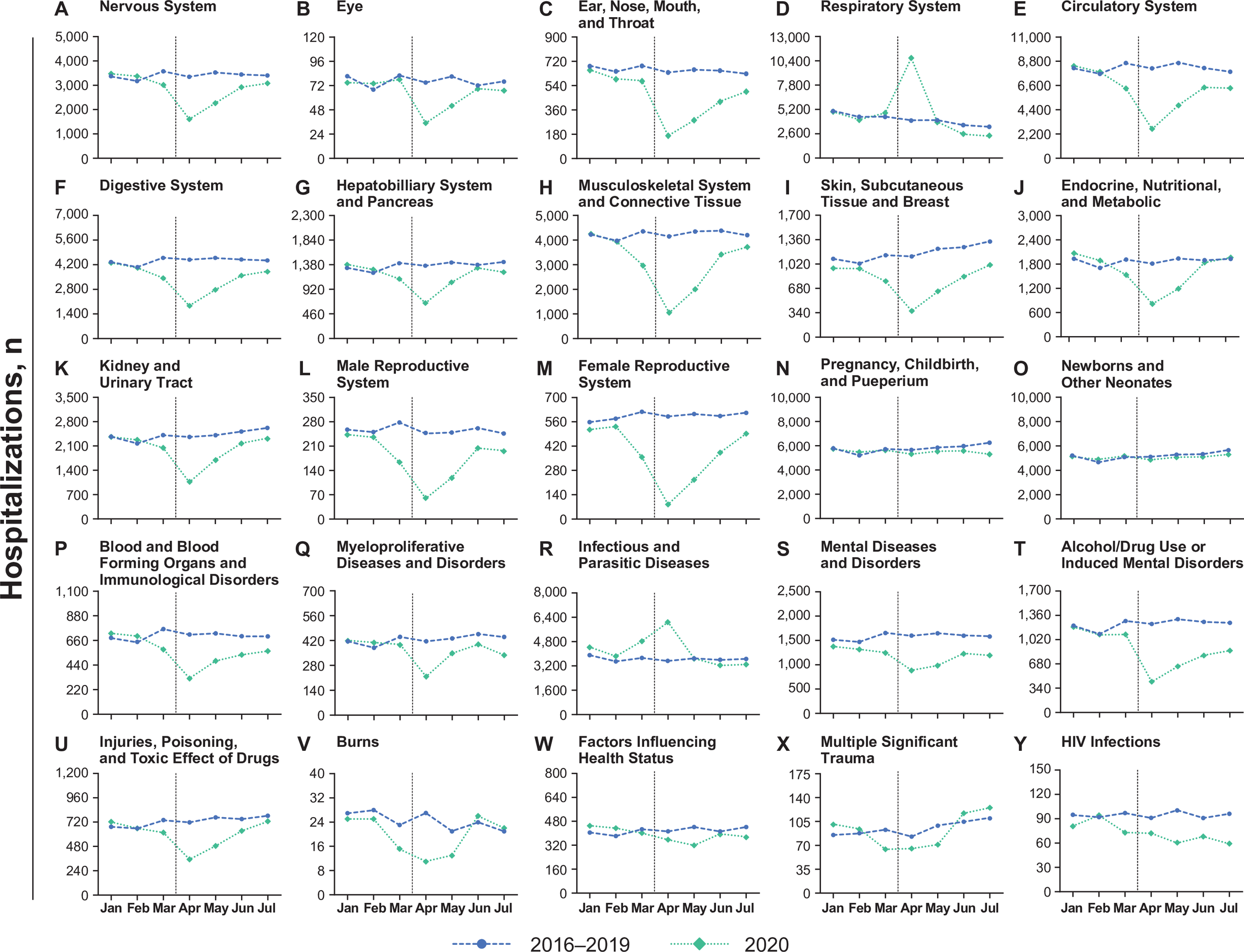

Supplement: S2 Fig — (TIF) [file pone.0262347.s002.tif]

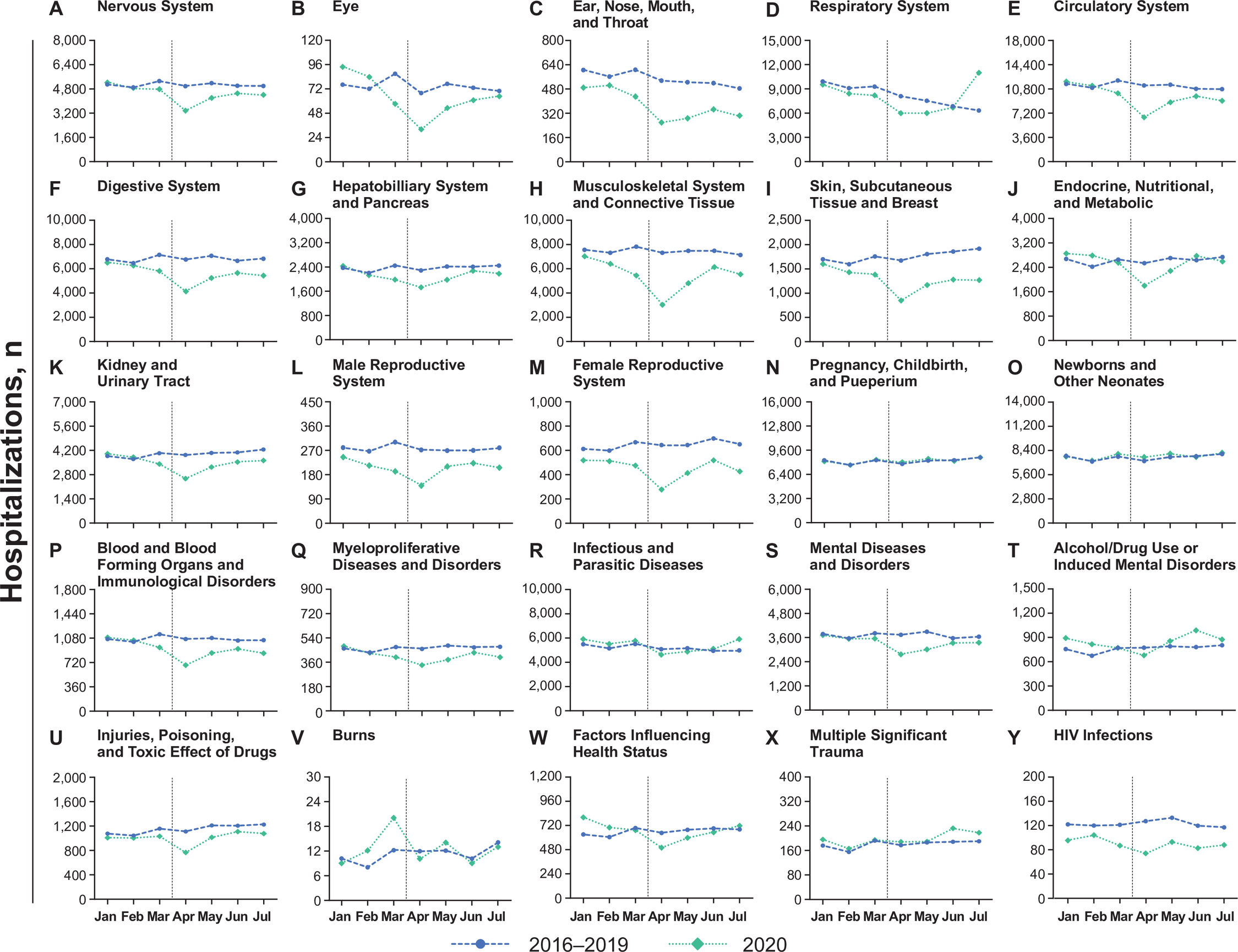

Supplement: S3 Fig — (TIF) [file pone.0262347.s003.tif]

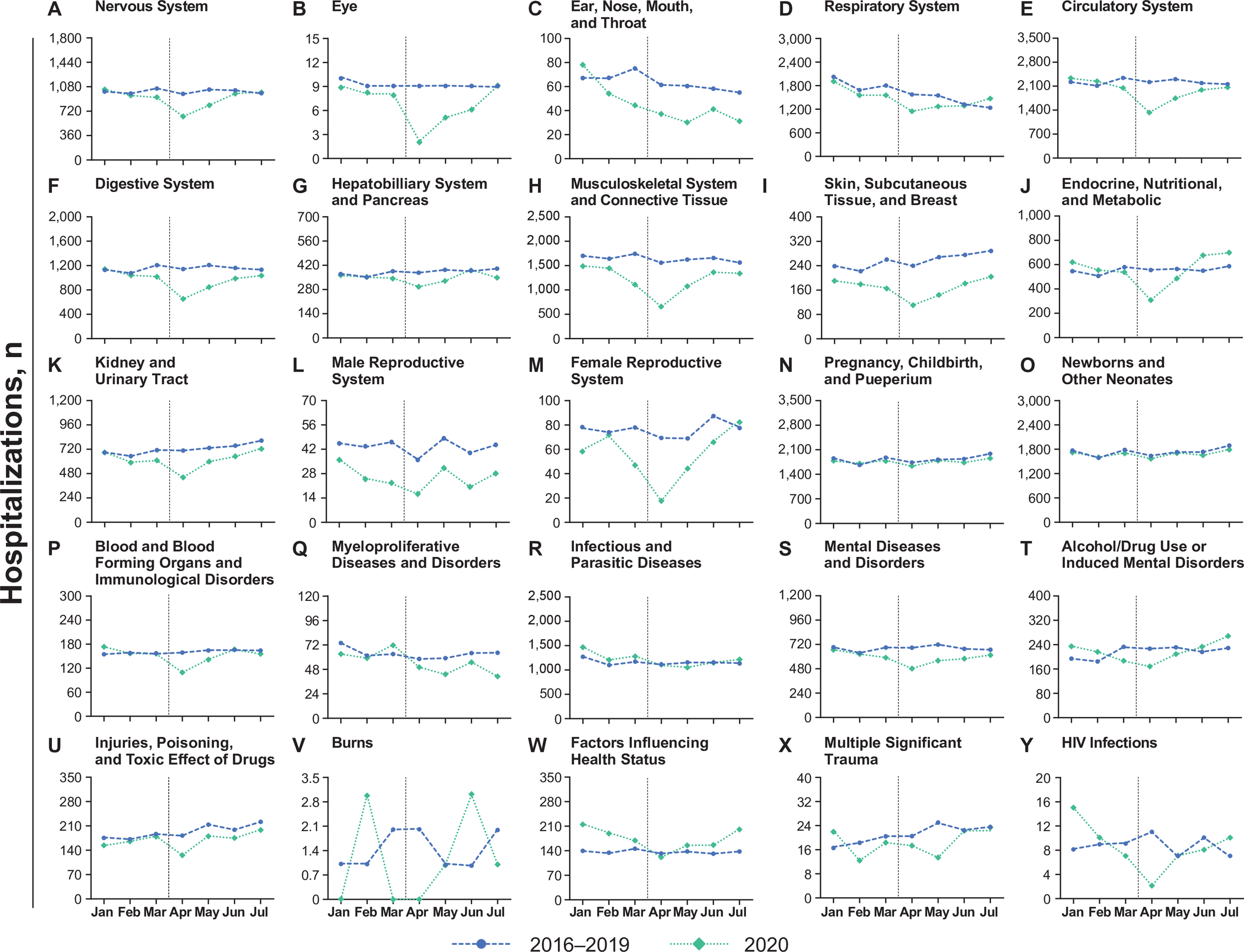

Supplement: S4 Fig — (TIF) [file pone.0262347.s004.tif]

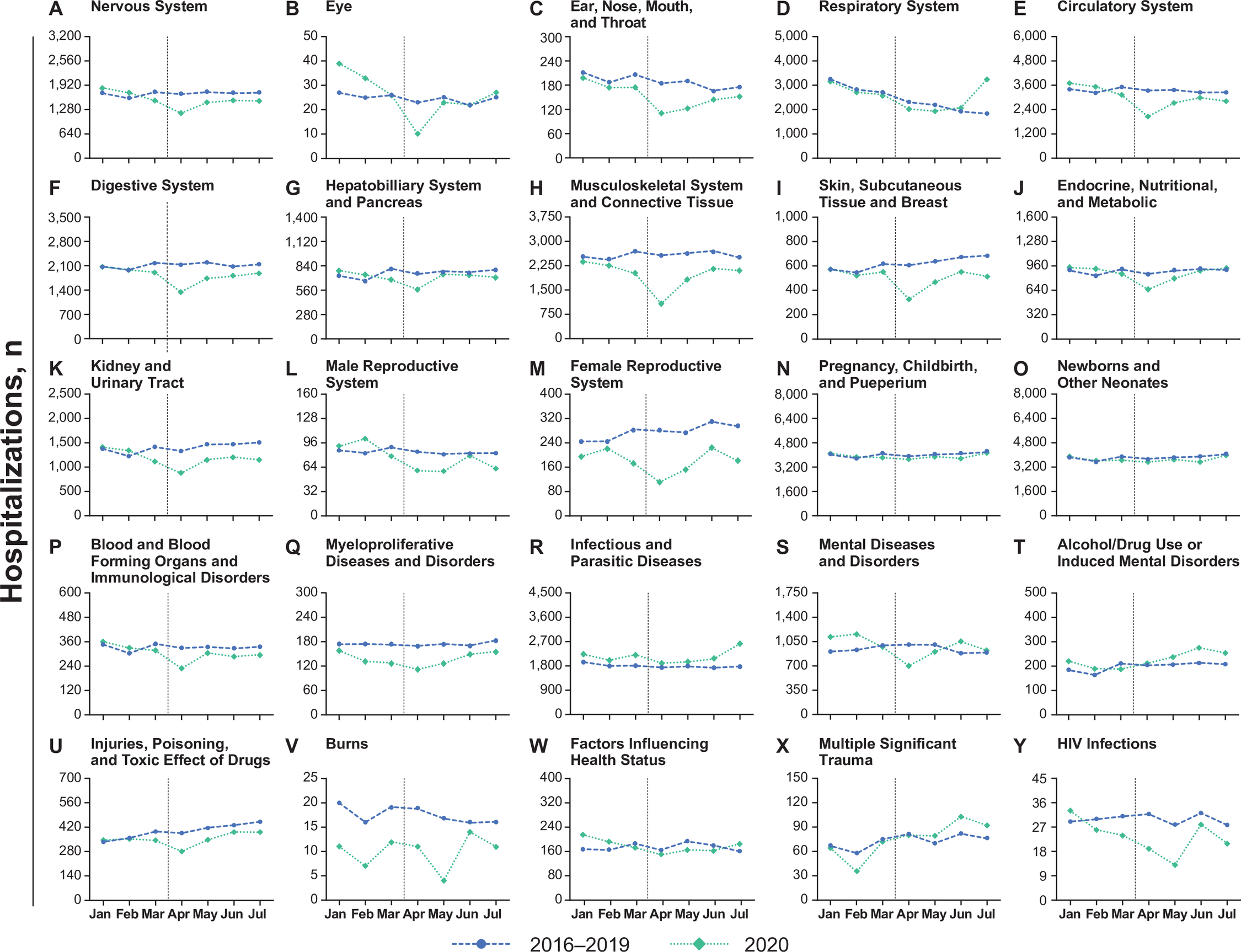

Supplement: S5 Fig — (TIF) [file pone.0262347.s005.tif]

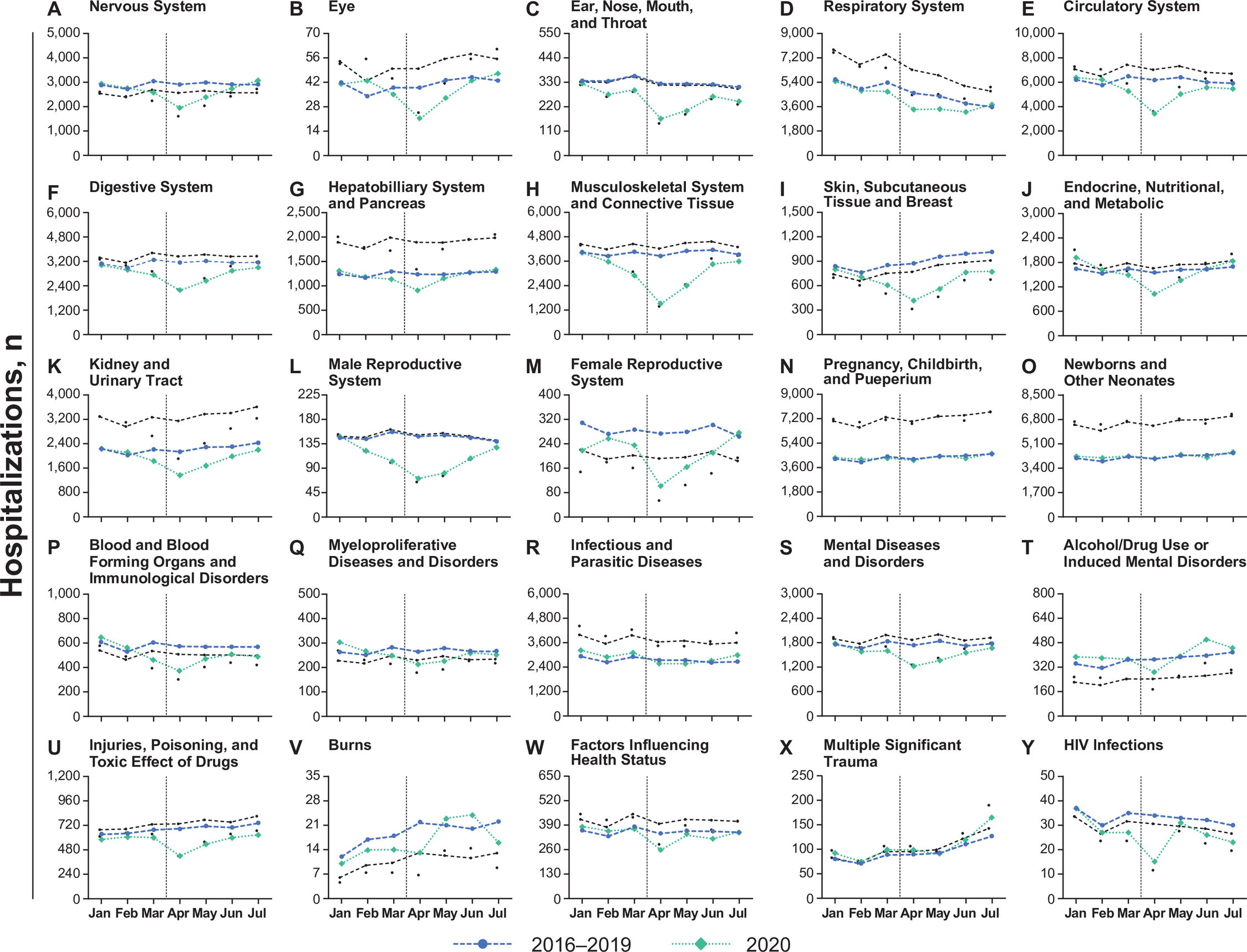

Supplement: S6 Fig — (TIF) [file pone.0262347.s006.tif]

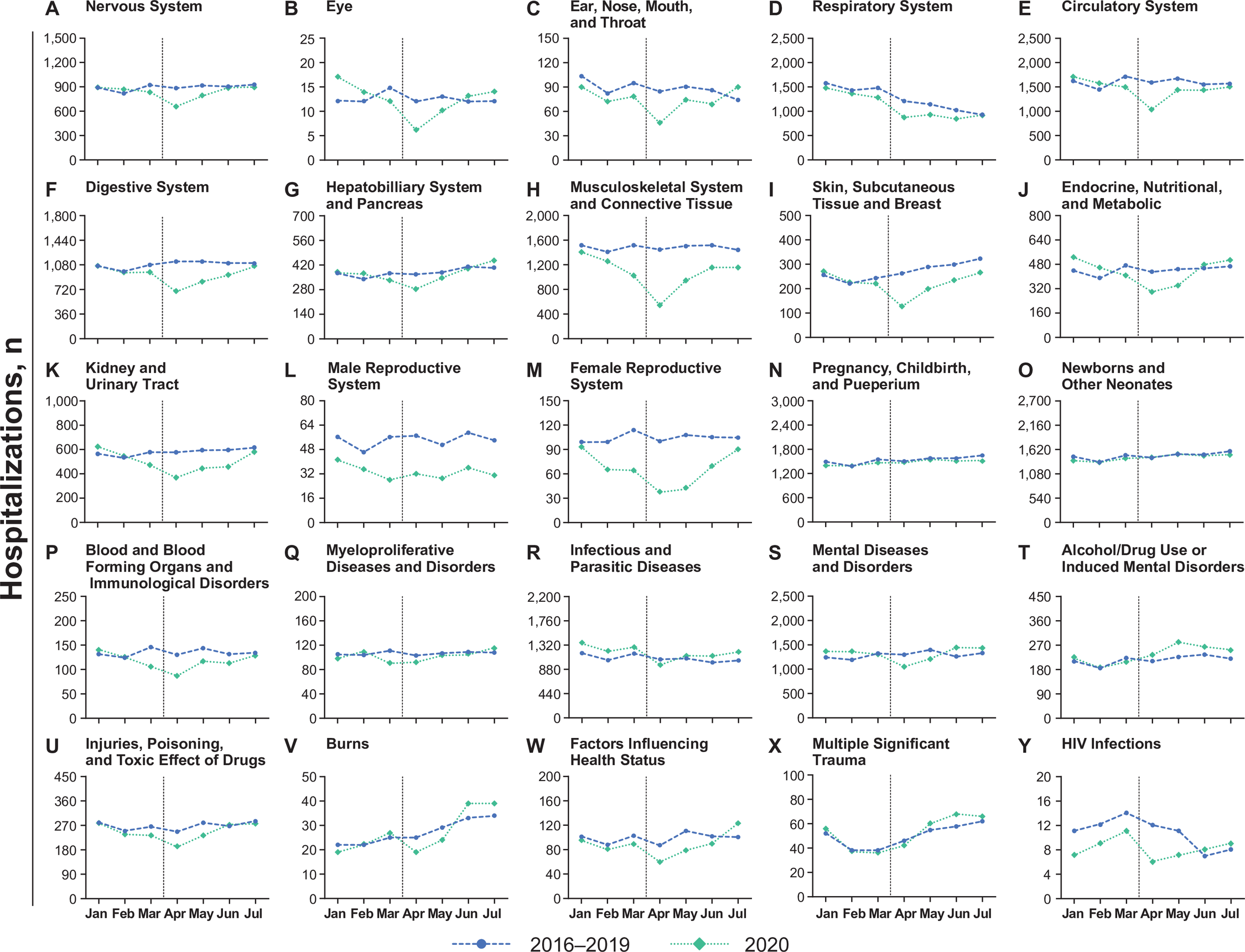

Supplement: S7 Fig — (TIF) [file pone.0262347.s007.tif]

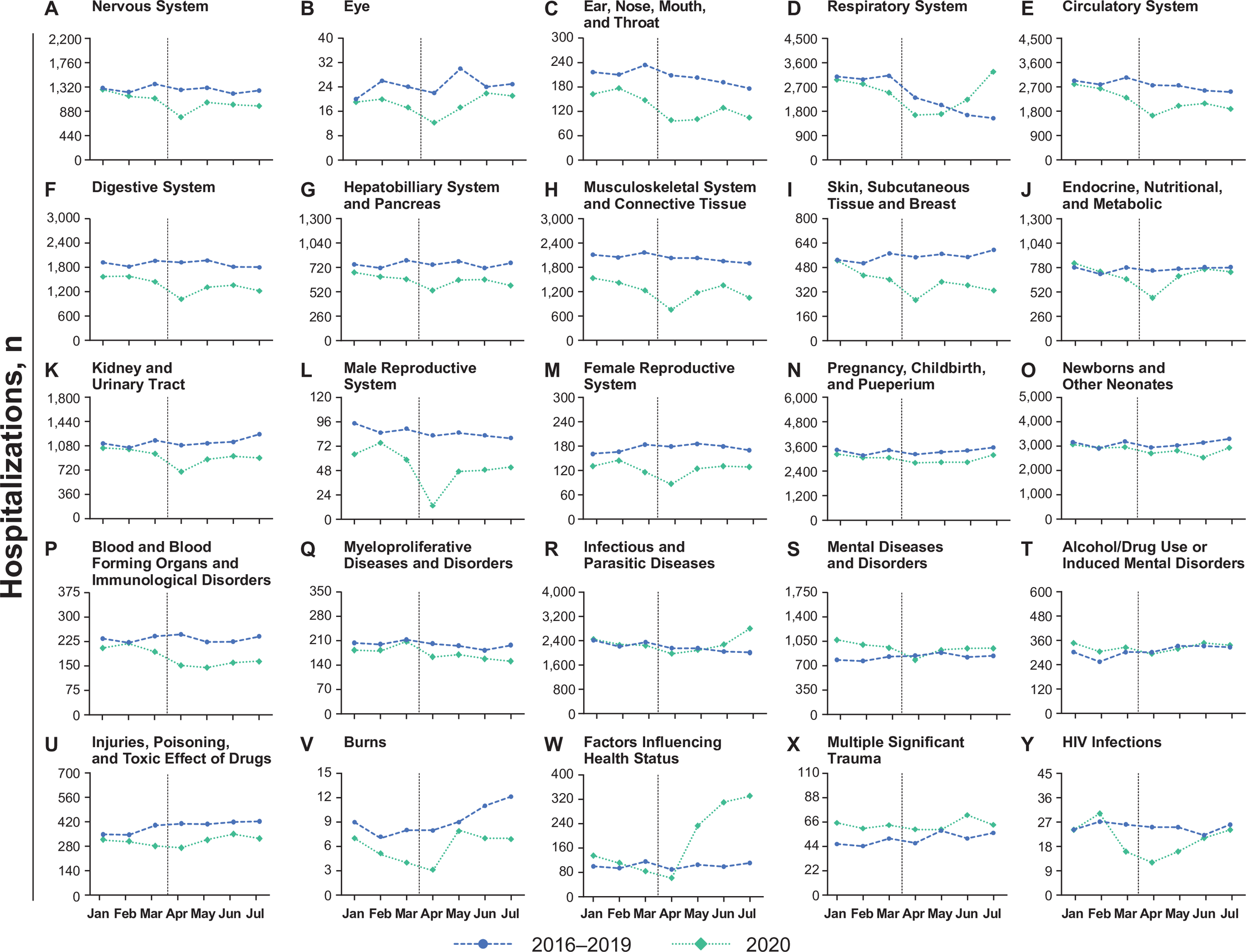

Supplement: S8 Fig — (TIF) [file pone.0262347.s008.tif]

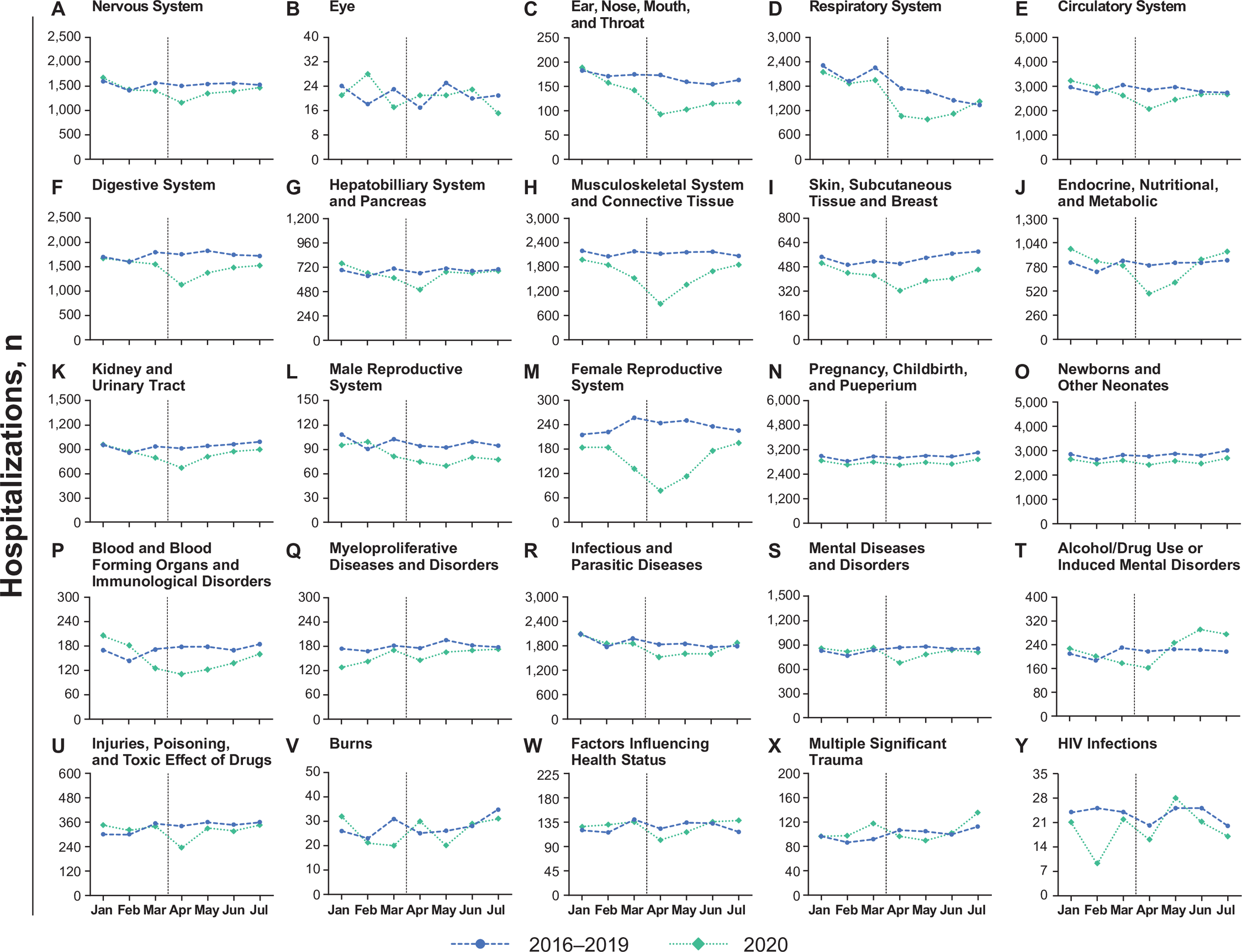

Supplement: S9 Fig — (TIF) [file pone.0262347.s009.tif]

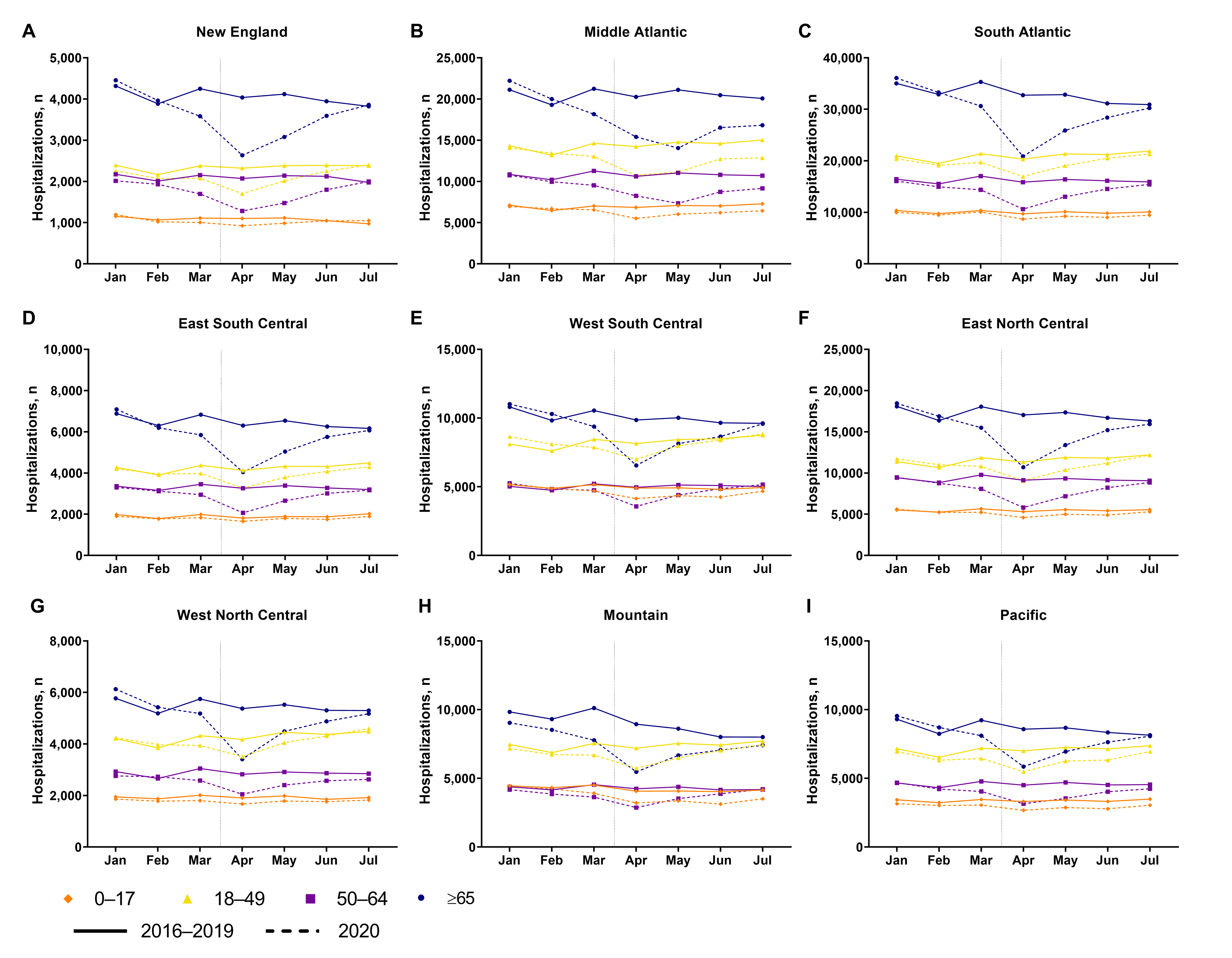

Supplement: S10 Fig — (TIF) [file pone.0262347.s010.tif]
