# Supplementary material for: Pandemic-related declines in hospitalization for non-COVID-19-related illness in the United States from January through July 2020
Source: PLoS One. 2022 Jan 6;17(1):e0262347. doi: 10.1371/journal.pone.0262347 (PMC8735608; doi:10.1371/journal.pone.0262347)
Supplement: S1 Table — *COVID-19 case counts were extracted from the COVID-19 Dashboard by the Center for Systems Science and Engineering at Johns Hopkins University [17]. (DOCX) [file pone.0262347.s011.docx]

**S1 Table. Average monthly hospitalizations, January–July 2016-2019 compared with monthly hospitalizations and monthly reported COVID-19 cases* in 2020, United States.**

|  | **2016–2019** | **2020** | **US COVID-19 Cases** | |
| --- | --- | --- | --- | --- |
| **All-Cause Hospitalizations** | | | | |
| January | 301,812 | 302,390 | 8 | |
| February | 279,804 | 280,411 | 17 | |
| March | 306,043 | 268,459 | 186,028 | |
| April | 288,456 | 211,178 | 881,268 | |
| May | 296,815 | 239,889 | 710,069 | |
| June | 288,921 | 261,032 | 823,659 | |
| July | 290,523 | 276,071 | 1,888,140 | |
| **Non-Respiratory System Hospitalizations** | | | | |
| January | 267,687 | 269,401 | 8 | |
| February | 249,331 | 251,687 | 17 | |
| March | 274,348 | 239,725 | 186,028 | |
| April | 261,390 | 182,892 | 881,268 | |
| May | 271,233 | 218,576 | 710,069 | |
| June | 266,363 | 240,132 | 823,659 | |
| July | 269,483 | 247,800 | 1,888,140 | |
| **Respiratory System and COVID-19 Hospitalizations** | | | | |
|  |  |  | **COVID-19 Hospitalizations** | **US COVID-19 Cases** |
| January | 34,125 | 32,989 |  | 8 |
| February | 30,473 | 28,754 |  | 17 |
| March | 31,695 | 28,734 |  | 186,028 |
| April | 27,066 | 28,286 | 21,944 | 881,268 |
| May | 25,582 | 21,313 | 12,058 | 710,069 |
| June | 22,558 | 20,900 | 9,448 | 823,659 |
| July | 21,040 | 28,271 | 19,961 | 1,888,140 |
