# Supplementary material for: Pandemic-related declines in hospitalization for non-COVID-19-related illness in the United States from January through July 2020
Source: PLoS One. 2022 Jan 6;17(1):e0262347. doi: 10.1371/journal.pone.0262347 (PMC8735608; doi:10.1371/journal.pone.0262347)
Supplement: S2 Table — (DOCX) [file pone.0262347.s012.docx]

**Supplementary Table 2. Average monthly hospitalizations, January–July, in 2016–2019 compared with monthly hospitalizations in 2020, by major diagnostic category, United States.**

|  | **January–July**  **2016–2019** | **January–July**  **2020** | **Total of Average Monthly Hospitalizations for January–July 2016–2019 vs January–July 2020**  ***P*-value** |
| --- | --- | --- | --- |
| Nervous System | | |  |
| January | 18,443 | 18,912 |  |
| February | 17,265 | 17,547 |  |
| March | 19,103 | 16,601 |  |
| April | 18,095 | 11,702 |  |
| May | 18,786 | 14,726 |  |
| June | 18,318 | 16,439 |  |
| July | 18,250 | 16,945 |  |
| Total* | 128,259 | 112,872 | <0.0001 |
| Eye | | |  |
| January | 302 | 329 |  |
| February | 271 | 307 |  |
| March | 312 | 255 |  |
| April | 272 | 142 |  |
| May | 309 | 223 |  |
| June | 286 | 262 |  |
| July | 289 | 270 |  |
| Total* | 2,041 | 1,788 | <0.0001 |
| Ear, Nose, Mouth and Throat | | |  |
| January | 2495 | 2256 |  |
| February | 2335 | 2063 |  |
| March | 2513 | 1947 |  |
| April | 2292 | 1001 |  |
| May | 2283 | 1242 |  |
| June | 2216 | 1567 |  |
| July | 2119 | 1586 |  |
| Total* | 16,253 | 11,662 | <0.0001 |
| Respiratory System | | |  |
| January | 34,125 | 32,989 |  |
| February | 30,473 | 28,754 |  |
| March | 31,695 | 28,734 |  |
| April | 27,066 | 28,286 |  |
| May | 25,582 | 21,313 |  |
| June | 22,558 | 20,900 |  |
| July | 21,040 | 28,271 |  |
| Total* | 192,539 | 189,247 | <0.0001 |
| Circulatory System | | |  |
| January | 40,234 | 41,570 |  |
| February | 37,803 | 39,398 |  |
| March | 41,993 | 34,338 |  |
| April | 39,567 | 21,415 |  |
| May | 40,709 | 29,861 |  |
| June | 38,426 | 33,946 |  |
| July | 37,853 | 32,805 |  |
| Total* | 276,584 | 233,333 | <0.0001 |
| Digestive System | | |  |
| January | 23,210 | 22,443 |  |
| February | 21,847 | 21,268 |  |
| March | 24,431 | 19,679 |  |
| April | 23,623 | 13,386 |  |
| May | 24,351 | 17,305 |  |
| June | 23,327 | 19,509 |  |
| July | 23,449 | 19,955 |  |
| Total* | 164,237 | 133,545 | <0.0001 |
| Hepatobiliary System and Pancreas | | |  |
| January | 8255 | 8496 |  |
| February | 7646 | 7715 |  |
| March | 8618 | 7147 |  |
| April | 8196 | 5707 |  |
| May | 8521 | 7253 |  |
| June | 8382 | 8052 |  |
| July | 8628 | 7942 |  |
| Total* | 58,245 | 52,312 | <0.0001 |
| Musculoskeletal System and Connective Tissue | | |  |
| January | 27,715 | 25,926 |  |
| February | 26,340 | 23,776 |  |
| March | 28,368 | 19,435 |  |
| April | 26,758 | 9942 |  |
| May | 27,609 | 16,345 |  |
| June | 27,775 | 22,346 |  |
| July | 26,354 | 22,153 |  |
| Total* | 190,918 | 139,923 | <0.0001 |
| Skin, Subcutaneous Tissue and Breast | | |  |
| January | 5954 | 5595 |  |
| February | 5532 | 5019 |  |
| March | 6151 | 4676 |  |
| April | 6000 | 2858 |  |
| May | 6507 | 4089 |  |
| June | 6688 | 4777 |  |
| July | 6972 | 5023 |  |
| Total* | 43,803 | 32,037 | <0.0001 |
| Endocrine, Nutritional, and Metabolic | | |  |
| January | 10,116 | 11,126 |  |
| February | 9148 | 10,139 |  |
| March | 10,193 | 9158 |  |
| April | 9618 | 6030 |  |
| May | 10,114 | 8006 |  |
| June | 10,060 | 10,282 |  |
| July | 10,324 | 10,613 |  |
| Total* | 69,572 | 65,354 | 0.0049 |
| Kidney and Urinary Tract | | |  |
| January | 13,578 | 13,735 |  |
| February | 12,594 | 12,919 |  |
| March | 13,851 | 11,617 |  |
| April | 13,439 | 8279 |  |
| May | 14,016 | 10,844 |  |
| June | 14,221 | 12,202 |  |
| July | 14,941 | 12,807 |  |
| Total* | 96,639 | 82,403 | <0.0001 |
| Male Reproductive System | | |  |
| January | 1095 | 986 |  |
| February | 1030 | 935 |  |
| March | 1143 | 754 |  |
| April | 1044 | 488 |  |
| May | 1046 | 669 |  |
| June | 1068 | 828 |  |
| July | 1041 | 804 |  |
| Total* | 7,465 | 5,464 | <0.0001 |
| Female Reproductive System | | |  |
| January | 2336 | 1957 |  |
| February | 2306 | 2048 |  |
| March | 2562 | 1641 |  |
| April | 2453 | 820 |  |
| May | 2480 | 1308 |  |
| June | 2585 | 1821 |  |
| July | 2453 | 1916 |  |
| Total* | 17,174 | 11,511 | <0.0001 |
| Pregnancy, Childbirth, and Puerperium | | |  |
| January | 33,016 | 32,419 |  |
| February | 30,397 | 30,701 |  |
| March | 33,226 | 32,012 |  |
| April | 31,808 | 30,440 |  |
| May | 33,188 | 32,118 |  |
| June | 33,431 | 31,402 |  |
| July | 35,034 | 33,099 |  |
| Total* | 230,101 | 222,191 | <0.0001 |
| Newborns and Other Neonates | | |  |
| January | 30,943 | 30,579 |  |
| February | 28,415 | 28,878 |  |
| March | 31,004 | 30,567 |  |
| April | 29,689 | 29,012 |  |
| May | 31,022 | 30,617 |  |
| June | 31,212 | 29,436 |  |
| July | 32,763 | 31,747 |  |
| Total* | 215,047 | 210,836 | 0.0014 |
| Blood and Blood Forming Organs and Immunological Disorders | | | |
| January | 3522 | 3663 |  |
| February | 3266 | 3419 |  |
| March | 3690 | 2989 |  |
| April | 3514 | 2136 |  |
| May | 3535 | 2735 |  |
| June | 3449 | 2938 |  |
| July | 3482 | 2933 |  |
| Total* | 24,457 | 20,813 | <0.0001 |
| Myeloproliferative Diseases and Disorders | | |  |
| January | 1912 | 1884 |  |
| February | 1801 | 1770 |  |
| March | 1982 | 1758 |  |
| April | 1886 | 1359 |  |
| May | 1970 | 1606 |  |
| June | 1940 | 1775 |  |
| July | 1955 | 1660 |  |
| Total* | 13,445 | 11,812 | <0.0001 |
| Infectious and Parasitic Diseases | | |  |
| January | 21,853 | 23,712 |  |
| February | 19,785 | 21,359 |  |
| March | 21,220 | 23,143 |  |
| April | 19,764 | 21,259 |  |
| May | 20,187 | 19,510 |  |
| June | 19,457 | 19,714 |  |
| July | 19,646 | 22,438 |  |
| Total* | 141,911 | 151,135 | 0.0006 |
| Mental Disease and Disorders | | |  |
| January | 11,910 | 12,398 |  |
| February | 11,374 | 11,785 |  |
| March | 12,419 | 11,515 |  |
| April | 12,182 | 8878 |  |
| May | 12,718 | 10,078 |  |
| June | 11,805 | 11,362 |  |
| July | 11,971 | 11,343 |  |
| Total* | 84,378 | 77,359 | <0.0001 |
| Alcohol/Drug Use or Induced Mental Disorders | | |  |
| January | 3585 | 3862 |  |
| February | 3223 | 3510 |  |
| March | 3799 | 3467 |  |
| April | 3703 | 2587 |  |
| May | 3878 | 3327 |  |
| June | 3831 | 3870 |  |
| July | 3852 | 3772 |  |
| Total* | 25,870 | 24,395 | 0.0242 |
| Injuries, Poisoning, and Toxic Effect of Drugs | | |  |
| January | 4008 | 3909 |  |
| February | 3927 | 3830 |  |
| March | 4344 | 3785 |  |
| April | 4258 | 2797 |  |
| May | 4555 | 3588 |  |
| June | 4501 | 4027 |  |
| July | 4664 | 4151 |  |
| Total* | 30,256 | 26,087 | <0.0001 |
| Burns | | |  |
| January | 127 | 113 |  |
| February | 123 | 111 |  |
| March | 139 | 112 |  |
| April | 140 | 98 |  |
| May | 137 | 108 |  |
| June | 145 | 152 |  |
| July | 155 | 141 |  |
| Total* | 964 | 835 | 0.0009 |
| Factors Influencing Health Status | | |  |
| January | 2089 | 2497 |  |
| February | 1975 | 2274 |  |
| March | 2262 | 2174 |  |
| April | 2063 | 1674 |  |
| May | 2233 | 2088 |  |
| June | 2166 | 2294 |  |
| July | 2166 | 2528 |  |
| Total* | 14,954 | 15,529 | 0.0136 |
| Multiple Significant Trauma | | |  |
| January | 634 | 709 |  |
| February | 577 | 595 |  |
| March | 661 | 681 |  |
| April | 668 | 661 |  |
| May | 712 | 674 |  |
| June | 733 | 864 |  |
| July | 783 | 914 |  |
| Total* | 4,769 | 5,098 | 0.1517 |
| HIV Infections |  |  |  |
| January | 360 | 325 |  |
| February | 355 | 321 |  |
| March | 367 | 274 |  |
| April | 360 | 221 |  |
| May | 367 | 256 |  |
| June | 347 | 267 |  |
| July | 339 | 255 |  |
| Total* | 2,494 | 1,919 | <0.0001 |

HIV=Human immunodeficiency virus infection and acquired immune deficiency syndrome.

*May not sum due to rounding.
