# Supplementary material for: Pandemic-related declines in hospitalization for non-COVID-19-related illness in the United States from January through July 2020
Source: PLoS One. 2022 Jan 6;17(1):e0262347. doi: 10.1371/journal.pone.0262347 (PMC8735608; doi:10.1371/journal.pone.0262347)
Supplement: S3 Table — (DOCX) [file pone.0262347.s013.docx]

**Supplementary Table 3. Average monthly all-cause hospitalizations, January–July, in 2016–2019 compared with monthly all-cause hospitalizations in 2020, by age group, United States.**

|  | **January–July 2016–2019** | **January–July**  **2020** | **Total of Average Monthly Hospitalizations for January–July 2016–2019 vs January–July 2020**  ***P*-value** |
| --- | --- | --- | --- |
| **Aged 0–17 years** |  |  |  |
| January | 41,146 | 40,212 |  |
| February | 38,639 | 38,108 |  |
| March | 41,285 | 38,171 |  |
| April | 39,014 | 33,091 |  |
| May | 40,187 | 35,466 |  |
| June | 39,226 | 34,848 |  |
| July | 40,433 | 37,200 |  |
| Total | 279,930 | 257,096 | <0.0001 |
| **Aged 18–49 years** |  |  |  |
| January | 80,280 | 79,654 |  |
| February | 74,258 | 74,711 |  |
| March | 82,175 | 74,531 |  |
| April | 78,835 | 63,449 |  |
| May | 82,410 | 71,169 |  |
| June | 81,763 | 76,847 |  |
| July | 84,311 | 80,893 |  |
| Total | 564,032 | 521,254 | <0.0001 |
| **Aged 50–64 years** |  |  |  |
| January | 59,262 | 58,515 |  |
| February | 55,625 | 54,366 |  |
| March | 61,281 | 51,619 |  |
| April | 57,473 | 39,701 |  |
| May | 59,422 | 45,555 |  |
| June | 58,115 | 51,651 |  |
| July | 57,445 | 54,830 |  |
| Total | 408,623 | 356,237 | <0.0001 |
| **Aged ≥65 years** |  |  |  |
| January | 121,124 | 124,009 |  |
| February | 111,281 | 113,256 |  |
| March | 121,301 | 104,138 |  |
| April | 113,134 | 74,937 |  |
| May | 114,796 | 87,699 |  |
| June | 109,818 | 97,686 |  |
| July | 108,334 | 103,148 |  |
| Total | 799,788 | 704,873 | <0.0001 |
